# Astaxanthin Protects against Hyperglycemia-Induced Oxidative and Inflammatory Damage to Bone Marrow and to Bone Marrow-Retained Stem Cells and Restores Normal Hematopoiesis in Streptozotocin-Induced Diabetic Mice

**DOI:** 10.3390/antiox11122321

**Published:** 2022-11-23

**Authors:** Govinda Bhattarai, Han-Sol So, Tae-Geum Kim, Thi Thu Trang Kieu, Yeon-Woo Kim, Ku-Ri Yang, Jeong-Chae Lee, Sung-Ho Kook, Young-Mi Jeon

**Affiliations:** 1 Cluster for Craniofacial Development and Regeneration Research, Jeonbuk National University, Jeonju 54896, Republic of Korea; 2 Institute of Oral Biosciences and School of Dentistry, Jeonbuk National University, Jeonju 54896, Republic of Korea; 3 Department of Bioactive Material Sciences, Research Center of Bioactive Materials, Jeonbuk National University, Jeonju 54896, Republic of Korea; 4 Department of Bio-Convergence Science, Jeongup Campus of Jeonbuk National University, Jeongup 56212, Republic of Korea; 5 Research Institute of Clinical Medicine of Jeonbuk National University, Jeonju 54907, Republic of Korea

**Keywords:** astaxanthin, hyperglycemic complications, bone marrow microenvironment, stem cell senescence, hematopoiesis, bone mass accrual

## Abstract

Hyperglycemia has various adverse health effects, some of which are due to chronic oxidative and inflammatory impairment of bone marrow (BM), hematopoietic stem cells (HSCs), and mesenchymal stem cells (MSCs). Astaxanthin (ASTX) has been shown to ameliorate hyperglycemia-associated systemic complications and acute mortality, and this effect is partially associated with restoration of normal hematopoiesis. Here, the effects of ASTX on diabetes-induced complications in BM and BM stem cells were investigated, and the underlying molecular mechanisms were elucidated. Ten-week-old C57BL/6 mice received a single intraperitoneal injection of streptozotocin (STZ; 150 mg/kg) in combination with oral gavage of ASTX (12.5 mg/kg) for 30 or 60 consecutive days. Supplemental ASTX ameliorated acute mortality and restored the STZ-impaired bone mass accrual and BM microenvironment in STZ-injected mice. Oral gavage of ASTX suppressed osteoclast formation in the BM of STZ-injected mice. Specifically, supplementation with ASTX inhibited oxidative stress and senescence induction of BM HSCs and MSCs and ameliorated hematopoietic disorders in STZ-injected mice. These effects of ASTX were associated with BM restoration of angiopoietin 1, stromal cell-derived factor 1, β-catenin, and Nrf2. Long-term ASTX gavage also recovered the STZ-induced dysfunction in migration, colony formation, and mineralization of BM-derived stromal cells. Further, a direct addition of ASTX exhibited direct and dose-dependent inhibition of osteoclastic activation without cytotoxic effects. Collectively, these results indicate that ASTX protects against diabetes-induced damage in the BM microenvironment in BM, HSCs, and MSCs and restores normal hematopoiesis and bone accrual in STZ-injected mice.

## 1. Introduction

Diabetes mellitus (DM) is a metabolic syndrome characterized by impaired insulin secretion, insulin sensitivity, or both [1]. DM causes chronic hyperglycemia, lipid abnormalities, and cardiovascular complications due to various oxidative and inflammatory abnormalities [2,3,4]. Chronic oxidative stress and inflammation alter physiological processes, damage biomolecules, and promote degenerative diseases [5,6]. DM also accelerates the fragility of bones and delays the processes required for wound healing [7,8].

Numerous studies have shown that long-term diabetic disorders are orchestrated by impairments in the bone marrow (BM) microenvironment and hematopoietic development [9,10,11,12]. BM is a specialized niche essential for the maintenance and self-renewal of hematopoietic stem cells (HSCs) and mesenchymal stem cells (MSCs) [12,13]. Streptozotocin (STZ)-induced type 1 diabetic mice exhibited impaired repopulation of Lin^-^Sca-1^+^c-Kit^+^ (LSK) cells in the BM [9,14]. In addition, a decreased number of circulating CD34^+^ cells, along with reduced mobilization of hematopoietic progenitor cells (HPCs) in diabetic patients, were found to be associated with DM-mediated hematopoietic complications [14,15]. STZ-induced diabetic mice showed dysregulated interactions between MSC-derived stromal cell-derived factor 1 (SDF-1) and HSC-expressed C-X-C chemokine receptor type 4 (CXCR4), resulting in poor peripheral mobilization of hematopoietic cells [14,16]. Together, the available evidence suggests that long-term hyperglycemic conditions impair the BM microenvironment, disrupt BM retention, impair the functions of MSCs and HSCs, and delay homeostatic bone reparation and regenerative tissue healing [17].

Interaction of HSCs with osteoblastic niche cells is important for their retention and development in BM. Osteoblastic cells secrete various cytokines and chemokines that support the survival and differentiation of BM HSCs [18]. In addition to SDF-1, osteoblastic cells produce angiopoietin 1 (Ang1), Wnt ligands, β-catenin, and Dickkopf-related protein 1 (DKK1), through which the cells control the processes required for angiogenesis, hematopoiesis, and osteogenesis [18,19,20]. Therefore, diabetic complications in the BM could disrupt the osteoblastic niche and produce subsequent damage of the self-renewal, homeostatic differentiation, and migration capabilities of hematopoietic cells. Furthermore, dysregulation of the Ang1/Tie2 axis, the SDF-1/CXCR4 axis, or both, might accompany diabetes-induced damage to the BM environment and to hematopoietic and mesenchymal stem cells in the bone.

Hyperglycemia-related local and systemic disorders have been attributed to prolonged oxidative and inflammatory damage to cells and tissues. Oxidative stress and inflammatory conditions in the BM are also hallmarks of impaired BM retention and senescence induction of hematopoietic and mesenchymal stem cells in the bone [2,3,9,10,11,12]. Clinical use of non-toxic and dietary antioxidants is considered a useful approach for treating diabetes [21,22]. Among numerous antioxidative molecules, astaxanthin (3,3′-dihydroxy-β,β′-carotene-4,4′-dione; ASTX) has been shown to have strong antioxidant [23] and anti-inflammatory [24,25] activity and to regulate positively the antioxidant defense systems [26,27,28]. ASTX has also been shown to have beneficial effects on DM-associated complications, as well as on aging, cancer, and degenerative pathologies in various organs [23]. It was recently reported that long-term oral gavage of ASTX significantly inhibited periodontal destruction, diminished oxidative complications, and improved nuclear factor erythroid-2-related factor 2 (Nrf2)-dependent activity in STZ-induced diabetic mice [29]. It was also found that supplemental ASTX did not directly improve hyperglycemia but attenuated STZ-induced periodontal destruction by ameliorating oxidative and inflammatory systemic complications [29]. Other studies have demonstrated that supplemental ASTX activates T-helper cells [30] ameliorate the redox imbalance in the lymphocytes of STZ-induced diabetic rats [22]. The previous findings, together with those of other studies, indicate that supplemental ASTX protects against diabetes-induced impairment of the BM microenvironment and hematopoietic system and can potentially be used to treat oxidative and inflammatory disorders in addition to its potential role in improving BM retention and preventing senescence induction of HSCs and MSCs in the BM. However, the precise roles of ASTX in diabetes-induced degenerative complications in the BM microenvironment and hematopoietic and mesenchymal stem cells in the bone remain unclear.

In this study, how supplemental ASTX affects diabetes-triggered complications in BM and BM stem cells was explored using a STZ-induced diabetic mouse model. To understand the cellular mechanisms by which supplemental ASTX protected against diabetic complications, the expression of various chemokines and signaling molecules involved in the regulation of homeostatic hematopoiesis and bone mass accrual was evaluated. The ex vivo effects of supplemental ASTX on migration, colony formation, and mineralization were also investigated using bone marrow stromal cells (BMSCs) derived from experimental mice. Additionally, the in vitro effects of ASTX on osteoclast formation, DNA damage, and viability were examined using primary cultured bone marrow monocytes (BMMs). Collectively, the current findings not only support a protective role for supplemental ASTX on diabetes-associated impairments in the hematopoietic system and BM microenvironment, but also provide further evidence of its clinical usefulness.

## 2. Materials and Methods

### 2.1. Reagents

ASTX (CAS No. 472-61-7) and STZ (CAS No. 18883-66-4) were purchased from Sigma-Aldrich Co. LLC (St. Louis, MO, USA), while fetal bovine serum (FBS) was obtained from HyClone Laboratories, Inc. (Logan, UT, USA). Antibodies specific to Nrf2 (BS1258) and receptor activator of nuclear factor (NF)-κB ligand (RANKL; ALX-804-243) were purchased from Bioworld Technology, Inc. (St. Louis Park, MN, USA) and Enzo Life Sciences, Inc. (Farmingdale, NY, USA), respectively. Antibodies specific to interleukin-1β (IL-1β; sc-52012), interferon-γ (IFN-γ; sc-57208), tumor necrosis factor α (TNF-α; sc-52746), and β-actin (sc-47778) were obtained from Santa Cruz Biotechnology (Santa Cruz, CA, USA). 2′,7′-Dichlorodihydrofluorescein-diacetate (DCF-DA), and anti-osterix (ab209484), anti-osteopontin (OPN; ab8448), anti-heme oxygenase 1 (HO-1; ab13248), anti-bone morphogenetic protein 2 (BMP2; ab214821), and anti-γ-H2AX (ab26350) antibodies were purchased from Abcam (Cambridge, UK). Unless otherwise specified, other reagents were purchased from Sigma-Aldrich Co. LLC, while laboratory consumables were from Falcon Labware (Becton-Dickinson, Franklin Lakes, NJ, USA).

### 2.2. Animals, Treatment, and Sample Preparation

Eight-week-old C57BL/6 male mice (total 210 heads) were supplied by Orient Bio (Daejeon, South Korea) and were randomly divided into five groups: non-diabetic control, vehicle, STZ, STZ+ASTX, and ASTX groups. Mice (*n* = 5/cage) were housed at 22 ± 1 °C and 55 ± 5% humidity on a 12 h light/dark auto-cycling with free access to food and water and monitored 12 h/day during the experimental period. At 2 weeks post-acclimatization, STZ and STZ+ASTX groups received STZ, as described previously [29]. Briefly, STZ was dissolved in sodium citrate buffer, and STZ and STZ+ASTX groups received a single intraperitoneal injection of 150 mg/kg STZ 6 h after fasting, while the control, vehicle, and ASTX groups were injected with buffer only. Blood glucose level was assessed 72 h after STZ injection using a digital glucometer (Accu-check^®^ Advantage, Roche Diagnostic, Mannheim, Germany); mice with a glucose level greater than 300 mg/dL were used as hyperglycemic diabetes mice in subsequent experiments. The STZ+ASTX group received 12.5 mg/kg ASTX via oral gavage in 100 µL olive oil (vehicle) daily for 30 or 60 consecutive days. The vehicle and STZ groups received 100 µL olive oil only. ASTX group received 12.5 mg/kg ASTX in 100 µL olive oil daily for 60 consecutive days without STZ injection. Blood glucose levels and body weight changes were monitored in all groups every 10 days after hyperglycemia induction. Consumption of water, food intake, and the survival rate of mice were observed daily. Experimental samples, including skeletal long bones, peripheral or whole blood, pancreas, and whole BM cells and BM supernatants were harvested 12 h after the final supplementation with ASTX.

### 2.3. Micro-Computed Tomography (µCT) Analysis

Femoral bones were scanned using a desktop scanner (1076 Skyscan Micro-CT; Skyscan, Kontich, Belgium) 60 days post hyperglycemia induction. Maximum voltage was 100 kV and maximum current was 100 µA using a 1-mm filter with a tomographic rotation of 360° (rotation step of 0.6°). Images were obtained at 18 µpixels, and data were analyzed using the SkyScan NRecon reconstruction package (Data viewer, Bruker-μCT-Analyzer version 1.13 and CT Vol, Bruker, Kontich, Belgium). Image slices were also reconstructed using cone-beam reconstruction software based on the Feldkamp algorithm (Dataviewer; Skyscan, Belgium). Values of bone-specific parameters, namely bone volume (BV, mm^3^), bone volume percentage (BV/TV, %), bone mineral density (BMD; g/cm^3^), trabecular number (Tb.N; 1/mm), and trabecular thickness (Tb.Th; mm) in trabecular and cortical regions of femoral bones were evaluated from the reconstructed 3D images, as described previously [31].

### 2.4. Hematoxylin and Eosin (H&E) Staining of Trabecular Bone

Femurs were collected from mice 60 days post-hyperglycemia induction, fixed in 4% paraformaldehyde solution for 48 h, and decalcified in 10% EDTA at 4 °C for 3 weeks. The tissues were dehydrated in an alcohol series, embedded in paraffin, and sectioned into slices of 5.0 µm thickness. Tissue sections were treated with hematoxylin solution (Gill No. 3) and counterstained with 0.25% Eosin Y stain (Thermo Fisher Scientific, Waltham, MA, USA). Finally, stained sections were observed under a light microscope (EL-Einsatz 451888, Carl Zeiss, Ostalbkreis, Germany).

### 2.5. Tartrate-Resistant Acid Phosphatase (TRAP) Staining of Femoral Bones

Osteoclasts formed in trabecular and cortical zones of femoral bones were quantified by staining tissue sections for TRAP using a leukocyte acid phosphatase kit (LOT# UIQ-04, Cosmo Bio, Tokyo, Japan). The TRAP-stained sections were counterstained with hematoxylin and photographed using a light microscope (EL-Einsatz 451888). Osteoclastic activation in the bones was determined by counting the number of TRAP^+^ osteoclasts formed in trabecular or cortical bone surface (mm^2^).

### 2.6. Immunohistochemistry (IHC)

Levels of HO-1 and Nrf2 in the pancreas were evaluated by IHC. Briefly, tissue sections were stained with primary antibody (1:200 dilution) specific to HO-1 or Nrf2. Expression patterns were determined using a mouse-anti-Vectasta in ABC DAB-HRP kit (LOT# ZH0901, Vector Laboratories, Burlingame, CA, USA), followed by observation under a light microscope (EL-Einsatz 451888). The area (%) of cells positively stained with HO-1 or Nrf2 was determined using ImageJ software (version 1.8.0, NIH, Bethesda, MD, USA). Unless otherwise specified, five mice per group were used for the staining assays, such as H&E, TRAP, and IHC, in which at least 5 tissue sections per mouse were analyzed in each assay.

### 2.7. Quantitative Reverse-Transcription Polymerase Chain Reaction (qRT-PCR) Assay

Total RNA was extracted from whole BM cells isolated from mice at 60 days post-hyperglycemia induction using TRIzol reagent (Invitrogen, Carlsbad, CA, USA). RNA samples (1 μg/sample) were subjected to cDNA synthesis using an AmpiGene cDNA synthesis kit (LOT# 10141515, Enzo Life Sciences, Inc.), following the manufacturer’s instruction. qRT-PCR was performed using Power SYBR Green PCR Master Mix (Applied Biosystems, Foster City, CA, USA) and an ABI StepOnePlus Real-Time PCR system (Applied Biosystems). Thermocycling conditions were 95 °C for 10 min for pre-denaturation, followed by denaturation at 95 °C for 15 s, annealing at 60 °C for 30 s, and extension at 72 °C for 30 s for 40 cycles. Oligonucleotide primers specific to SDF-1, Ang1, β-catenin, CXCR4, and DKK1 were designed (Table S1). Glyceraldehyde 3-phosphate dehydrogenase (*Gapdh*) was used as the endogenous control.

### 2.8. Flow Cytometric Analysis

Hematopoietic and mesenchymal stem/stromal cells in the BM were harvested from long bones of control, STZ, and STZ+ASTX mice at 30 or 60 days post-hyperglycemia induction. In brief, the ends of femoral and tibial bones were cut and flushed with phosphate-buffered saline (PBS) using a 5-mL syringe without crushing bones or treating with collagenase. BM cells were collected by centrifugation (1000× *g*) for 10 min and processed for the removal of red blood cells (RBCs). After washing with PBS, cells were analyzed by multicolor flow cytometry (BD Aria III, BD Biosciences, Franklin Lakes, NJ, USA), and phenotypical identification of cell populations was performed using FlowJo software (FLOWJO, Ashland, OR, USA) at the Center for University-Wide Research Facilities of Jeonbuk National University, as described previously [31]. In this study, BM LSK cells, Lin^−^Sca-1^−^c-Kit^+^ cells (hematopoietic progenitor cells, HPCs), and CD150^+^CD48^−^LSK cells (HSCs) were phenotypically identified after staining with lineage markers following phycoerythrin (PE)-Cy7-conjugated anti-CD3, anti-CD4, anti-CD8, anti-CD45R, anti-CD11b, anti-Gr-1, and anti-TER-119. In addition, cells were stained with PE- or fluorescein isothiocyanate (FITC)-conjugated anti-stem cell antigen 1 (Sca-1), an allophycocyanin (APC)-conjugated anti-c-kit, PerCP/Cy5.5-conjuated anti-CD150 (eBioscience, Waltham, MA, USA), and APC-Cy7-conjugated anti-CD48. MSCs (CD29^+^CD105^+^LSK) were characterized using a PE-Cy7-conjugated lineage cocktail, APC-Cy7-conjugated anti-Sca-1, PE-conjugated anti-CD29, and APC-conjugated anti-CD105 antibodies. Flow cytometric analysis was also carried out to assess oxidative stress, DNA damage, or senescence-related phenotypes in BM HSCs and MSCs. Briefly, mitochondrial superoxide anion level and senescence-associated β-galactosidase (SA-β-gal) activity was assessed with kits containing MitoSox Red (LOT# 2521059, Invitrogen, Carlsbad, CA, USA) or C_12_FDG (LOT# 0027, Invitrogen) according to the manufacturers’ instructions. Levels of cell cycle regulatory factor p16^INK4a^ (Santa Cruz Biotechnology) and the DNA damage marker γ-H2AX (Cell Signaling Technology, Danvers, MA, USA) in hematopoietic and mesenchymal stem/stromal cells were determined using Alexa Fluor 488-conjugated and PE-conjugated antibodies, respectively, after fixation and permeabilization. Alternatively, intracellular expression levels of Nrf2 in BM cells were measured by flow cytometry. To this end, whole BM cells isolated from femur and tibial bones were fixed, permeabilized, and stained with PE-conjugated Nrf2 antibody (#14409; Cell Signaling), followed by flow cytometric analysis. Here, cell fixation and permeabilization for intracellular staining were performed using BD Cytofix/Cytoperm^TM^ (Cat. No. 554714, BD Biosciences), according to the manufacturer’s instructions. Unless specified otherwise, other antibodies used in flow cytometric assay were purchased from BD Biosciences.

### 2.9. Counting of Circulating Blood Cells

Peripheral and whole blood samples were collected from mouse groups via tail vein cutting and cardiac puncture into K_2_EDTA-treated tubes (BD Biosciences), respectively, 30 and 60 days after hyperglycemia induction. Levels of circulating granulocytes, lymphocytes, RBC, and white blood cells (WBC) were analyzed using an automated blood cell counter (Sysmex XE-2100; TOA Medical Electronics Co., Kobe, Japan).

### 2.10. Measurement of RANKL in Serum

Serum was obtained from whole blood samples at 30 or 60 days after hyperglycemia induction by centrifuging whole blood samples at 1500× *g* in serum separator tubes. Serum level of RANKL was measured using a mouse-anti-RANKL ELISA Kit (LOT# GR3407600-1, Abcam) using a microplate reader (SPECTROstar^®^ Nano, BMG LABTECH, Ortenberg, Germany), following the manufacturers’ instructions.

### 2.11. Ex Vivo and In Vitro Assays of BM-Derived Cells

#### 2.11.1. Isolation and Culture of BMSCs and BMMs

Whole BM cells were isolated from long bones of mouse groups 60 days post-hyperglycemia induction. BM cells were collected by centrifugation for 10 min before the removal of RBCs. After washing with PBS, BM cells were resuspended in α-minimum essential media (α-MEM, Thermo Fisher Scientific, Waltham, MA, USA) and centrifuged at 2000× *g* for 3 min. Pellets were resuspended, seeded onto 60-mm tissue culture plates, and incubated in growth medium (α-MEM supplemented with 2 mM glutamine, 100 IU/mL penicillin G, 100 μg/mL streptomycin, and 20% FBS). After a 24-h incubation, non-adherent suspended cells were carefully removed, and adherent cells were collected by centrifugation at 2000× *g* for 5 min. Here, the adherent cells were used as BMSCs for ex vivo experiments. BMMs were also obtained from the long bones of 10-week-old B6 mice that did not receive STZ or ASTX treatment. Briefly, RBC-free BM cells were incubated for 24 h in growth medium and, then, non-adherent and suspended cells were harvested and collected to use as BMMs.

#### 2.11.2. Ex Vivo Assays to Assess BMSC Migration, Colony Formation, and Mineralization

The effect of supplemental ASTX on the migration of BMSCs into wound areas was initially evaluated on culture plates. In brief, control-, STZ-, and STZ+ASTX-derived BMSCs were spread onto 12-well culture plates (10^6^ cells/well). When these cells reached approximate 70% confluence, the bottoms of the plates were scraped to create a 1-mm-wide wound area. Twenty-four hours after the wound area was created, photographs were taken using an optical microscope (EL-Einsatz 451888), and wound areas (relative area (%) of migrated cells to that of control) were calculated using ImageJ software. BMSCs were also spread onto 6-well culture plates (10^6^ cells/well) in growth medium. After 14 days of incubation, adherent cells were fixed in 10% formalin for 10 min and stained with 0.5% crystal violet dissolved in 100% methanol. BMSC-derived colonies (CFU-F) containing more than 50 cells per colony were counted. In addition, BMSCs isolated from mouse groups were incubated in osteogenic medium supplemented with 5% FBS, 100 nM dexamethasone, 50 µM of ascorbic acid, and 10 mM β-glycerophosphate (DAG). Culture media was changed every 3 days throughout the incubation period. After 21 days of incubation, the degree of mineralization was determined by staining cells with Alizarin Red S after fixation for 30 min in 4% paraformaldehyde. Cells were stained with the red dye and photographed under a light microscope (EL-Einsatz 451888). The stained cells were also treated with 10% acetylpyridinum chloride, and the amount of red dye was quantified by measuring the dye-specific absorbance at 405 nm using a microplate reader (SPECTROstar^®^ Nano).

#### 2.11.3. Immunoblot Assay

BMSCs were spread onto 6-well culture plates (2 × 10^6^ cells/well) in growth medium. After 24 h of incubation, culture medium was changed to DAG-supplemented medium followed by an additional incubation for 5 days. At the end of the incubation, whole protein lysates were extracted from BMSC cultures, and extracts (20 μg/sample) were separated by sodium dodecyl sulfate-polyacrylamide gel electrophoresis on 12% gels. After electroblotting onto polyvinylidene difluoride membranes, blots were washed with buffer (10 mM Tris-HCl (pH 7.6), 150 mM NaCl, and 0.05% Tween-20) and treated with 5% skim milk for 1 h followed by incubation with primary OPN, osterix, BMP2, or β-actin antibodies (1:1000 or 2000 dilution). Blots were exposed to horseradish peroxidase-conjugated secondary antibodies, and immunoreactive bands were visualized by enhanced chemiluminescence (ELPIS-Biotech, Daejeon, Korea), followed by X-ray film exposure (Eastman Kodak, Rochester, NY, USA).

#### 2.11.4. TRAP Staining and qRT-PCR of BMMs

BMMs were spread onto 12-well culture plates (2 × 10^6^ cells/well) in α-MEM supplemented with 5% FBS, 30 ng/mL monocyte-colony stimulating factor (M-CSF) and 50 ng/mL RANKL in the presence or absence of ASTX (30 or 60 μM). During the incubation, medium was replaced with fresh medium every 3 days. After 7 days of incubation, BMMs were stained with TRAP, and TRAP-positive multinucleated cells (MNCs) were counted as osteoclasts. BMMs cultured for 7 days were also permeabilized with Triton^™^ X-100 and stained with FITC-conjugated phalloidin (green) to visualize the formation of F-actin rings. In addition, BMMs were incubated in osteoclastic medium with and without ASTX (30 or 60 μM). After 2 days of incubation, expression levels of osteoclastogenic molecules such as nuclear factor of activated T-cells, cytoplasmic 1 (*NFATc1*), *TRAP*, and cathepsin K (*CTSK*) were evaluated on qRT-PCR. All procedures for PCR followed the same methods described above. Primer sequences of the osteoclastic molecules are shown in Table S1.

#### 2.11.5. Assays for DNA Damage and Viability of Cultured BMMs

To evaluate the direct effects of ASTX on DNA and BMM viability, BMMs were seeded onto 6-well (2 × 10^6^ cells/well) or 96-multiwell culture plates (2 × 10^3^ cells/well) in growth medium supplemented with 30 or 60 μM ASTX. After 48 h of incubation, the level of γ-H2AX, a well-known marker of DNA double-strand breaks, was detected by flow cytometry using PE-conjugated antibodies. Viability of BMMs incubated in the same medium for 2 days was determined using a Cell Counting Kit-8 (LOT# DV684, CCK-8; Dojindo Lab, Rockville, MD, USA), according to the manufacturer’s instructions.

### 2.12. Statistical Analyses

Unless otherwise specified, all results are expressed as mean ± standard deviations. One-way ANOVA was applied to determine the significant differences among more than two experimental groups using SPSS (version 12.0, IBM, Chicago, IL, USA). Scheffe’s multiple range test was used for multiple comparisons among groups. The significance of differences between two groups was analyzed by unpaired Student’s *t*-tests. A *p* values less than 0.05 was considered statistically significant.

## 3. Results

### 3.1. Oral Supplementation with ASTX Diminishes Severe Mortality, but Not Hyperglycemic Condition, in STZ-Induced Diabetic Mice

**Figure 1:**
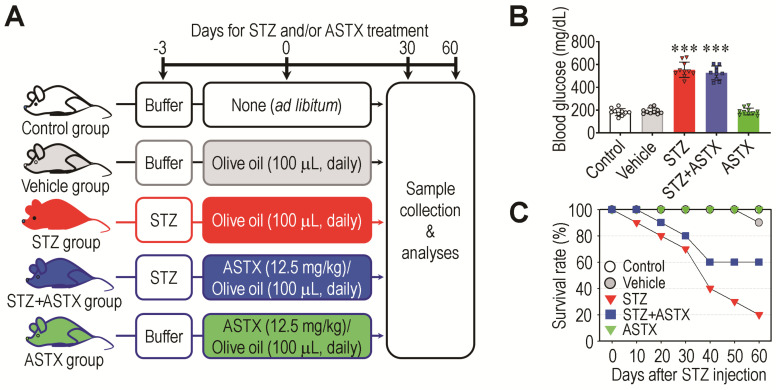
Long-term oral gavage of ASTX ameliorates hyperglycemia-induced lethality, but not blood glucose level, in STZ-injected mice. (**A**) A schematic illustration of the experimental design. (**B**) Blood glucose level of mouse groups at 60 days post-hyperglycemia induction (*n* = 7/group). (**C**) Survival rate (%) of mouse groups at the indicated days after STZ injection (*n* = 10/group). *** *p* < 0.001 by unpaired Student’s *t*-test.

A schematic diagram of the experimental design, including mouse groups, treatments, and schedules for sample collection, is shown in Figure 1A. Similar to previous findings [29], oral gavage of ASTX did not significantly ameliorate STZ-mediated complications, such as high blood glucose level (Figure 1B) or decreased body weight (Figure S1), even with long-term supplementation for 60 consecutive days. While survival rates in STZ and STZ+ASTX groups decreased in a time-dependent manner after STZ injection compared with the control group, the STZ group exhibited relatively more acute and severe mortality than did the STZ+ASTX group (Figure 1C). The vehicle and ASTX groups revealed survival rate (%) comparable with that of the control group. The STZ group exhibited a higher frequency of polydipsia and polyuria than the STZ+ASTX group (data not shown). These results indicate that long-term oral gavage of ASTX does not directly improve hyperglycemia, but ameliorates acute systemic complications in STZ-induced diabetic mice.

### 3.2. Oral Supplementation with ASTX Ameliorates the Impaired BM Microenvironment and Bone Mass Accrual in STZ-Induced Diabetic Mice

**Figure 2:**
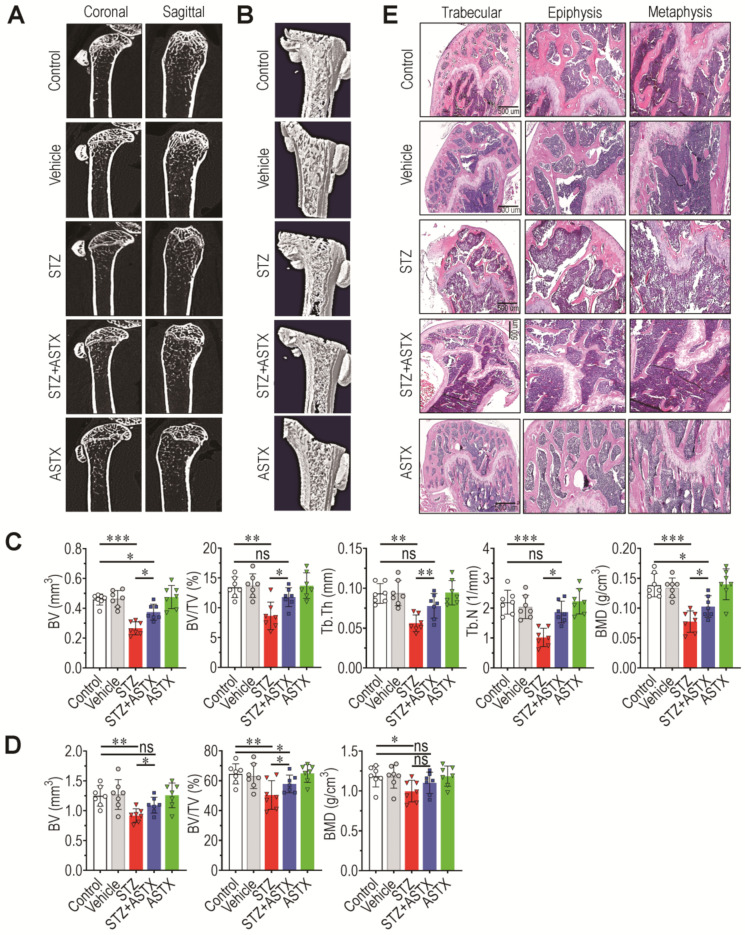
Long-term administration of ASTX ameliorates BM impairment in STZ-injected mice. (**A**) Representative 2D µCT images of coronal and sagittal sections of femoral bones at 60 days post-hyperglycemia induction. (**B**) Reconstructed three-dimensional µCT images of femoral bones. Based on the three-dimensional μCT reconstruction, bone parameter values in (**C**) trabecular and (**D**) cortical regions were calculated (*n* = 7/group). (**E**) H&E staining showing bone mass accrual in the trabecular bone of mice 60 days after hyperglycemia induction. Bar = 500 μm. * *p* < 0.05, ** *p* < 0.01, and *** *p* < 0.001 by unpaired Student’s *t*-test. ns, not significant.

It was examined whether hyperglycemic conditions actually impaired the BM microenvironment and bone mass accrual. Two-dimensional (2D) images (Figure 2A) and reconstructed three-dimensional μCT images (Figure 2B) indicated that cortical bone thickness and trabecular bone mass in the STZ group were relatively lower than those in control, vehicle, STZ-ASTX, or ASTX groups. Based on the three-dimensional images, STZ group had significantly lower BV, BV/TV, Tb.Th, Tb.N, and BMD values in trabecular (Figure 2C) and cortical regions (Figure 2D) than did the control or STZ+ASTX groups. Specifically, STZ-induced bone mass loss was more severe in trabecular than cortical bone. STZ-induced reduction of trabecular bone mass and its suppression by ASTX were also observed after H&E staining; the STZ group exhibited a severe decrease in both epiphysis and metaphysis compared with these processes in the control or STZ+ASTX groups (Figure 2E). All values of the bone parameters in the control group were not differed from those in vehicle or ASTX group. These results indicate that STZ-mediated hyperglycemia causes bone mass loss and impairs the BM microenvironment, and that these complications are recoverable via ASTX supplementation. These results also indicate that, in addition to hyperglycemic condition and survival rate, oral gavage of olive oil or ASTX itself for 60 consecutive days did not change BM microenvironment that is the critical factor in providing stem cell-specific niches.

### 3.3. Long-Term Supplementation with ASTX Ameliorates STZ-Induced Formation of Osteoclasts and Production of RANKL

**Figure 3:**
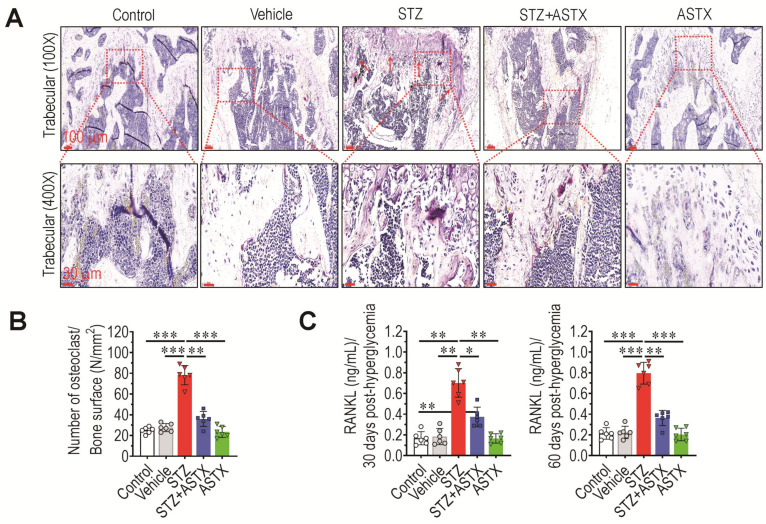
Oral supplementation with ASTX inhibits hyperglycemia-mediated osteoclast formation and RANKL secretion in the BM and serum of STZ-injected mice. (**A**) TRAP staining showing osteoclast formation in trabecular region of femoral bones at 60 days post-hyperglycemia induction. Red arrows indicate TRAP-positive osteoclasts. (**B**) Numbers of TRAP^+^ osteoclasts in trabecular region were determined as N/bone surface (mm^2^) using ImageJ (*n* = 5/group). (**C**) RANKL level in the serum of the five mouse groups was determined by ELISA 30 or 60 days after hyperglycemia induction (*n* = 5/group). * *p* < 0.05, ** *p* < 0.01, and *** *p* < 0.001 by unpaired Student’s *t*-test.

As decreased bone mass accrual is associated with skewed differentiation of progenitor cells toward osteoclasts, whether STZ-induced hyperglycemia promotes osteoclastic activation in long bones was evaluated. As shown in Figure 3A, the STZ group had more TRAP^+^ regions in trabecular bone than did control, vehicle, STZ+ASTX, or ASTX group. Significantly higher numbers of TRAP^+^ osteoclasts were found in the trabecular region of the STZ group compared with that of control (*p* < 0.001), vehicle (*p* < 0.001), STZ+ASTX (*p* < 0.01), or ASTX group (*p* < 0.001) (Figure 3B). Cortical region of the STZ group also revealed significantly higher formation of TRAP^+^ osteoclasts compared with that of other groups (data not shown). In addition, the STZ group exhibited higher concentrations of RANKL, a ligand for the receptor RANK, in serum than did the control, vehicle, STZ+ASTX, or ASTX groups both 30 and 60 days post-hyperglycemia induction (Figure 3C). There was no significant difference of RANKL between the control and STZ+ASTX groups at 60 days post-hyperglycemia induction.

### 3.4. Long-Term Supplementation with ASTX Increases the Induction of Nrf2 and HO-1 in STZ-Induced Diabetic Mice

**Figure 4:**
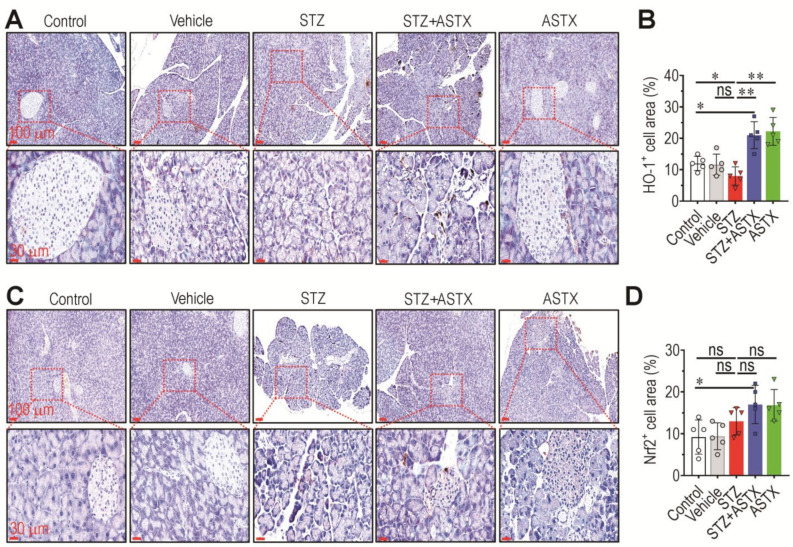
Oral supplementation with ASTX increases levels of HO-1 and Nrf2 in the pancreas of STZ-induced diabetic mice. Expression levels of (**A**) HO-1 and (**C**) Nrf2 in the pancreas of mouse groups were determined by IHC 60 days after hyperglycemia induction. The area (%) positively stained with (**B**) HO-1 or (**D**) Nrf2 was determined using ImageJ software (*n* = 5/group). * *p* < 0.05 and ** *p* < 0.01 by unpaired Student’s *t*-test. ns, not significant.

Nrf2 is a master transcriptional regulator of cellular antioxidant systems [28] and plays a crucial role in preventing and protecting against oxidative damage. The current findings, along with those of a previous study [29], strongly indicate that Nrf2 protects against diabetes-induced complications. To further clarify the role of the antioxidant defense system in STZ-induced diabetic mice, the levels of Nrf2 and its downstream effector, HO-1 in the pancreas of mouse groups, were determined at 60 days post-hyperglycemia induction. While level of HO-1 in STZ group tended to be reduced compared with the control group, this reduction was completely recovered by ASTX supplementation (Figure 4A). As shown in the IHC images, long-term treatment with ASTX also protected against the destruction of pancreatic islets, at least in part. When HO-1 cell area (%) of pancreatic sections was determined, the area was significantly higher in the STZ+ASTX or ASTX group than the control (*p* < 0.05) or STZ group (*p* < 0.01) (Figure 4B). In addition, both IHC images and cell area determination indicated that, unlike in HO-1, STZ injection itself did not alter protein levels of Nrf2 in the pancreas, whereas supplemental ASTX significantly increased its induction compared with the control group (Figure 4C,D).

### 3.5. Supplemental ASTX Inhibits STZ-Induced Oxidative Stress and Senescence of BM HSCs and Recovers Hematopoietic Disorders in STZ-Injected Mice

**Figure 5:**
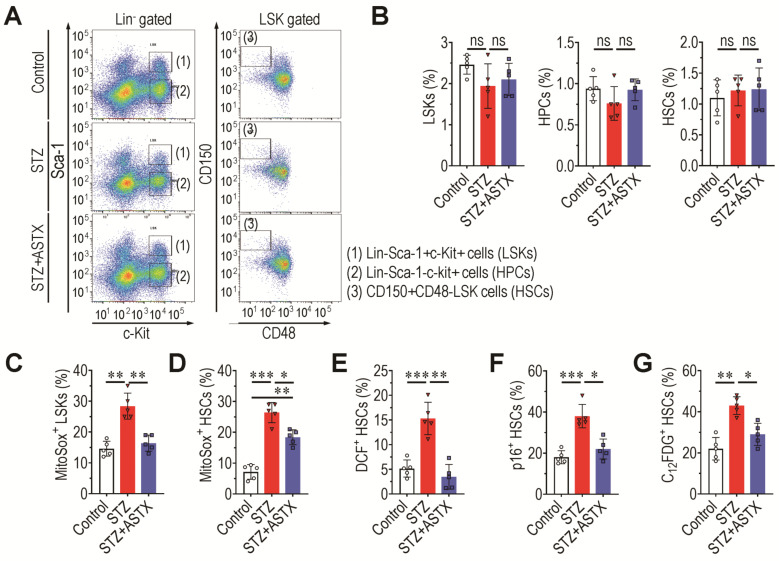
Supplemental ASTX attenuates hyperglycemia-induced oxidative stress and senescence in BM HSCs of STZ-injected mice. (**A**) BM frequencies of LSK cells, HPCs, and HSCs were analyzed by flow cytometry using specific markers. Representative results from five experiments are shown. (**B**) Mean percentages of these hematopoietic cells were calculated (*n* = 5/group). Levels of mitochondrial superoxide anions in (**C**) BM LSK cells and (**D**) HSCs, along with (**E**) BM levels of DCF^+^ HSCs in the mouse groups, were assessed by flow cytometry using MitoSox^TM^ Red or DCF-DA reagent at 60 days after hyperglycemia induction (*n* = 5/group). (**F**,**G**) Levels of senescent HSCs in the BM of the mouse groups were also measured by flow cytometry using p16^INK4a^ and C_12_FDG on the same post-hyperglycemia day (*n* = 5/group). * *p* < 0.05, ** *p* < 0.01, and *** *p* < 0.001 by unpaired Student’s *t*-test. ns, not significant.

BM microenvironment provides a niche for the maintenance and self-renewal of BM HSCs and MSCs, and its impairment leads to abnormal BM retention and development of these stem cells [32]. Long-term hyperglycemia may impair the BM microenvironment by increasing oxidative damage and senescence induction in BM stem cells. Therefore, the effects of supplemental ASTX on BM-retained stem cells were subsequently evaluated. As the vehicle and ASTX groups did not show significant differences with the control group in BM microenvironment and osteoclastic activation, the next experiments were also carried out using three mice groups such as the control, STZ, and STZ+ASTX groups. Figure 5A exhibits the BM frequencies (%) of LSK cells, HPCs, and HSCs in mouse groups at 60 days post-hyperglycemia induction via flow cytometric analysis. Average percentages of these cells in the STZ group were not different from those in the control or STZ+ASTX groups (Figure 5B). However, the BM frequencies (%) of LSK cells (Figure S2A and Figure 5C) and HSCs positively stained with MitoSox (Figure S2B and Figure 5D), along with DCF^+^ HSCs (Figure S2C and Figure 5E), were significantly higher in the STZ group than in the control group (*p* < 0.01 or *p* < 0.001) or STZ+ASTX group (*p* < 0.05 or *p* < 0.01). The BM level of senescent HSCs was also evaluated in mice by determining the percentages of p16^INK4a+^ and C_12_FDG^+^ HSCs 60 days after hyperglycemia induction. The STZ group exhibited approximately 2.1- and 1.7-fold higher levels of p16^INK4a+^ HSCs than the control and STZ+ASTX groups, respectively (Figure 5F). Similarly, the STZ group had a higher percentage of C_12_FDG^+^ HSCs than did the control (*p* < 0.01) or STZ+ASTX group (*p* < 0.05) (Figure 5G). These results indicate that STZ-induced diabetes causes oxidative stress and senescence induction in BM HSCs, and that this is recoverable by long-term supplementation with ASTX.

**Figure 6:**
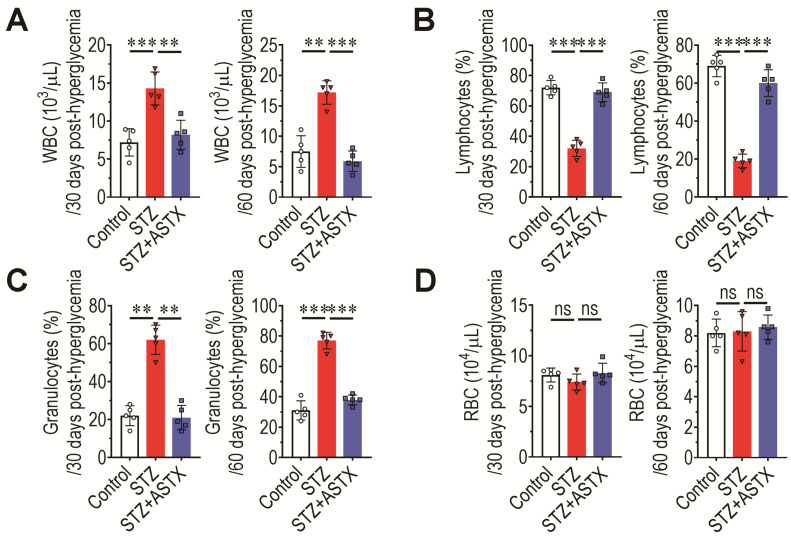
Oral gavage of ASTX restores normal production of circulating blood cells in STZ-injected mice. Levels of circulating (**A**) WBCs, (**B**) lymphocytes, (**C**) granulocytes, and (**D**) RBCs in mouse groups were measured using an automated complete blood cell counter at 30 and 60 days after hyperglycemia induction (*n* = 5/group). ** *p* < 0.01 and *** *p* < 0.001 by unpaired Student’s *t*-test. ns, not significant.

Impaired BM retention and senescence induction of HSCs contribute to abnormal hematopoietic development. Therefore, the levels of circulating blood cells in mice groups were determined at 30 and 60 days post-hyperglycemia induction. The STZ group exhibited an approximately two-fold higher level of WBCs 30 days after hyperglycemia induction than the control or STZ+ASTX group, and this difference was also markedly observed after 60 days (Figure 6A). Percentage (%) of circulating lymphocytes in the STZ group was significantly lower (*p* < 0.01 or *p* < 0.001) than that in the control or STZ+ASTX group both 30 and 60 days after hyperglycemia induction (Figure 6B). Similar to the WBC level, the percentage of granulocytes in the STZ group was increased compared with that in the control or STZ+ASTX groups (Figure 6C). However, the level of circulating RBCs (Figure 6D) was not affected by STZ injection with or without long-term oral gavage of ASTX, regardless of the number of days post-hyperglycemia. These results indicate that ASTX protects mice against STZ-induced hematopoietic defects, and that this is closely associated with its ability to inhibit oxidative stress and senescence induction of BM HSCs.

### 3.6. Long-Term Supplementation with ASTX Suppresses STZ-Induced Complications in BM Retention and Senescence Induction of MSCs

**Figure 7:**
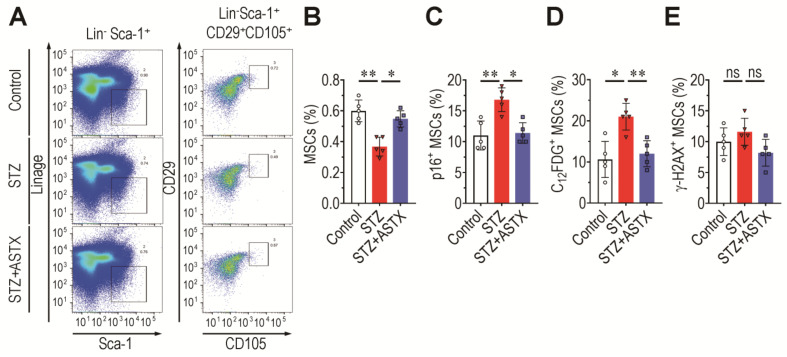
Supplemental ASTX inhibits hyperglycemia-mediated impairments in the BM MSCs of STZ-injected mice. (**A**) BM frequency of MSCs was determined by flow cytometry 60 days post-hyperglycemia induction, and (**B**) the mean percentage of these cells in mouse groups was calculated using FlowJo software (*n* = 5/group). (**C**,**D**) Levels (%) of senescent MSCs in the BM of mouse groups were assessed using the senescence markers p16^INK4a^ and C_12_FDG on the same post-hyperglycemia day by flow cytometry (*n* = 5/group). (**E**) γ-H2AX^+^ MSC level in the BM of mouse groups was also measured by flow cytometry on the same post-hyperglycemia day (*n* = 5/group). * *p* < 0.05 and ** *p* < 0.01 by unpaired Student’s *t*-test. ns, not significant.

MSCs interact with HSCs in the BM niche and play critical roles in maintaining the BM microenvironment and bone mass accrual [33]. If STZ-induced disorders are also related to impaired retention and senescence induction of BM, MSCs were investigated. In this study, BM MSCs were phenotypically defined as Lin^−^, Sca-1^+^, c-Kit^+^, CD105^+^, and CD29^+^ cells. Flow cytometric histograms showing the signal intensity of MitoSox or DCF indicated that expression of oxidative stress markers in BM MSCs was not changed by STZ injection in combination with ASTX (Figure S3A). The mean percentage of MitoSox^+^ or DCF^+^ MSCs also indicated that STZ, ASTX, or both had no effect on the induction of oxidative stress in BM-derived MSCs (Figure S3B,C). However, BM frequency (%) of MSCs in the STZ group was significantly lower than that in the control (*p* < 0.01) or STZ+ASTX group (*p* < 0.05) (Figure 7A,B). The STZ group also exhibited approximate 1.5- and two-fold higher percentages of p16^INK4a+^ (Figure 7C) and C_12_FDG^+^ MSCs (Figure 7D) than the control group, respectively. The STZ-induced increases in these senescence markers in BM MSCs were significantly (*p* < 0.05 or *p* < 0.01) diminished by long-term oral gavage of ASTX (Figure 7A–D). However, the level of γ-H2AX in BM MSCs did not differ among mouse groups at 60 days post-hyperglycemia induction (Figure 7E). These results indicate that STZ-induced diabetes induces impaired BM retention and senescence of MSCs without direct DNA damage, and that this is ameliorated by long-term ASTX gavage.

### 3.7. Long-Term Hyperglycemia Disturbs Ang1-, SDF-1-, and Wnt-Associated Signaling Pathways in the BM

**Figure 8:**
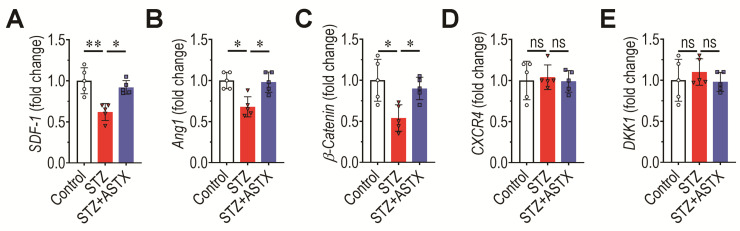
Supplemental ASTX restores mRNA expression of SDF-1, Ang1, and β-catenin in the BM of STZ-injected mice. Expression levels of (**A**) *SDF-1*, (**B**) *Ang1*, (**C**) *β-catenin*, (**D**) *CXCR4*, and (**E**) *DKK1* in whole BM cells were determined at 60 days post-hyperglycemia induction by qRT-PCR (*n* = 5/group). * *p* < 0.05 and ** *p* < 0.01 by unpaired Student’s *t*-test. ns, not significant.

Signaling pathways, such as the Ang1/Tie2, Wnt/β-catenin, and SDF-1/CXCR4 signaling pathways, play important roles in regulating the BM microenvironment, the fate of BM stem cells, and bone mass accrual. The qRT-PCR was performed to explore how STZ-induced diabetes affected the expression of several key molecules. STZ injection reduced significantly mRNA expression of SDF-1 (*p* < 0.01; Figure 8A), Ang1 (*p* < 0.05; Figure 8B), and β-catenin (*p* < 0.05; Figure 8C) in BM cells at 60 days post-hyperglycemia induction. As shown in these figures, the reduced expression of these molecules was mostly recovered by ASTX supplementation. However, in BM cells, mRNA levels of CXCR4, a receptor of SDF-1, and DKK1, a negative regulator of Wnt/β-catenin signaling pathway, were not altered by STZ injection with or without ASTX compared with the untreated control group (Figure 8D,E). These results may suggest that hyperglycemia affects the induction of ligands that activate specific signaling pathways, whereas further detailed experiments are required.

### 3.8. Long-Term Gavage of ASTX Recovers STZ-Induced Dysfunction of BMSC Migration, Colony Formation, and Mineralization

**Figure 9:**
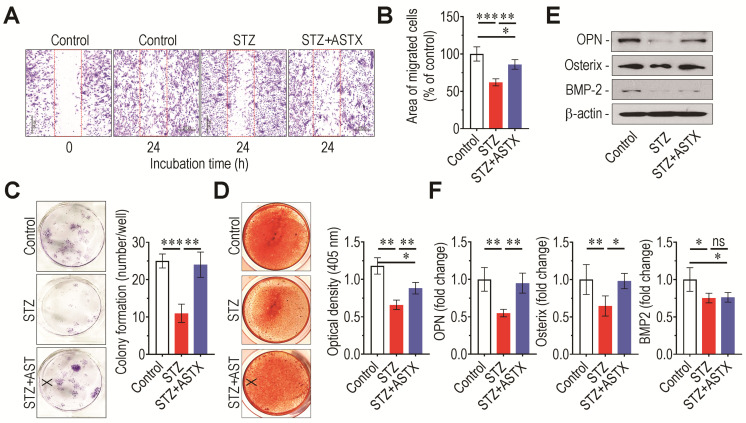
Oral supplementation with ASTX restores ex vivo migration, colony formation, and osteogenic potentials of BMSCs derived from STZ-injected mice. BMSCs derived from mouse groups were cultured on 12-well culture plates in growth medium, and wound areas were created by scraping cells. (**A**) After 24 h of incubation, wound areas were photographed (Bar = 500 μm), and (**B**) the area covered by migrated cells was calculated as a percentage of the control (*n* = 5/group). (**C**) Colonies formed by BMSCs that had been incubated in growth medium for 14 days were photographed, and colony numbers were counted under a light microscope (*n* = 5/group). BMSCs derived from the mouse groups were also incubated in DAG-supplemented osteogenic medium. (**D**) After 21 days of incubation, cells were stained with Alizarin Red S, and the red dye-specific optical density of cells was determined using a microplate reader (*n* = 5/group). (**E**) Protein levels of osteogenic markers in these cells were analyzed by immunoblot assay five days after incubation. (**F**) Band intensities specific to OPN, osterix, and BMP2 were calculated using ImageJ software (*n* = 5/group). * *p* < 0.05, ** *p* < 0.01, and *** *p* < 0.001 by unpaired Student’s *t*-test.

Next, the role of supplemental ASTX on STZ-induced diabetic complications in stem cells was investigated via ex vivo experiments. Compared with control- or STZ+ASTX group-derived BMSCs, STZ group-derived BMSCs showed significantly lower migration into wound areas on culture plates (Figure 9A,B). Similarly, STZ group-derived BMSCs formed approximate 11 colonies/well, and colony formation was significantly (*p* < 0.01) augmented by up to more than two-fold in control- and STZ+ASTX-derived BMSCs (Figure 9C). STZ group-derived BMSCs also had apparently lower mineralization in DAG-supplemented cultures than BMSCs derived from control (*p* < 0.001) or STZ+ASTX (*p* < 0.01) group (Figure 9D). In parallel with this, immunoblot assay indicated that supplemental ASTX restored the osteogenic potential of BMSCs in STZ-injected mice via restoration of osterix and OPN rather than BMP2 (Figure 9E,F). Together, these results demonstrate that long-term gavage of ASTX restores the stem-like potential of BMSCs under hyperglycemic conditions.

### 3.9. Direct Addition of ASTX Inhibits Osteoclastic Activation of BMMs in a Dose-Dependent Manner but Does Not Promote DNA Damage and Proliferation

**Figure 10:**
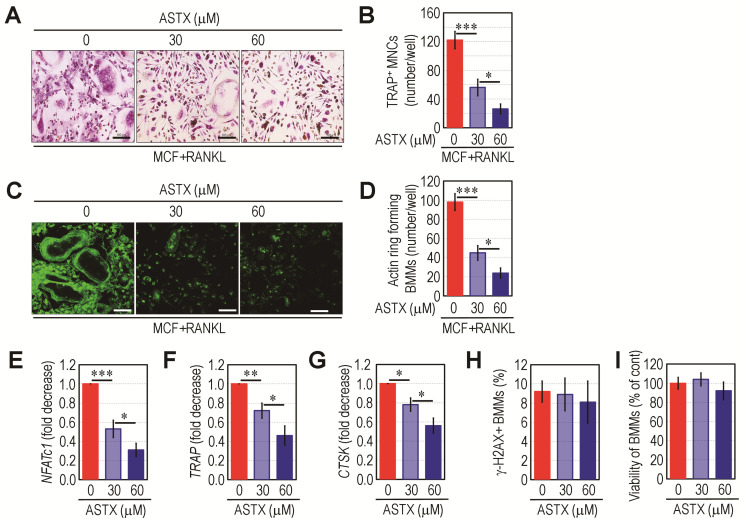
Direct addition of ASTX decreases the formation of osteoclasts and F-actin rings in BMMs in a dose-dependent manner. B6 mice-derived BMMs were incubated in osteoclastogenic medium supplemented with M-CSF and RANKL with or without 30 or 60 μM ASTX. (**A**) After seven days of incubation, cells were stained with TRAP (Bar = 100 μm) and (**B**) the number of TRAP^+^ MNCs was calculated under a light microscope. (**C**,**D**) On the same post-incubation day, the formation of F-actin rings in these cells was observed under a fluorescence microscope (Bar = 100 μm), and the number of BMMs with actin ring formation was counted. Levels of (**E**) *NFATc1*, (**F**) *TRAP*, and (**G**) *CTSK* in the cultured BMMs were determined by qRT-PCR two days after incubation. Additionally, BMMs were incubated in growth medium in the presence and absence of the indicated amounts of ASTX for two days. (**H**) Level of γ-H2AX^+^ cells and (**I**) the viability of these cells were determined using a flow cytometer or microplate reader after staining with a DNA damage marker or CCK-8 assay kit. * *p* < 0.05, ** *p* < 0.01, and *** *p* < 0.001 by Scheffe’s multiple range test.

It was previously found that the BMMs derived from STZ group showed greater osteoclast forming activity than did the cells from control or STZ+ASTX group [29]. To further understand the role of ASTX in osteoclastogenesis, BMMs were incubated in M-CSF- and RANKL-supplemented medium plus or minus 30 or 60 μM ASTX. Addition of ASTX suppressed the formation of TRAP^+^ MNCs from BMMs in a dose-dependent manner (Figure 10A,B). Parallel formation of F-actin rings in MNCs was significantly attenuated by ASTX treatment (Figure 10C,D). The potential of ASTX to inhibit osteoclast formation from M-CSF and RANKL-exposed BMMs was closely associated with its ability to downregulate the expression of *NFATc1*, *TRAP*, and *CTSK* (Figure 10E–G). However, ASTX treatment at the indicated concentrations did not increase the percentages (%) of γ-H2AX^+^ BMMs (Figure 10H) or CCK-8^+^ BMMs (Figure 10I). These results indicate that ASTX has direct and dose-dependent anti-osteoclastic effects without any side effects on DNA strand breakage or the viability of BMMs.

## 4. Discussion

ASTX exists naturally in seafood with a red-orange xanthophyll carotenoid [22]. FDA approved the use of ASTX as a food additive in 1999. Because of its strong potentials on antioxidation and anti-inflammation, numerous studies have highlighted its clinical usefulness on chronic oxidative and inflammatory diseases [4,22,23,24,25,26,27,28,29,30]. A report showed the effect of ASTX similar to that of monacolin K in STZ-induced diabetic rats, thereby indicating the use of ASTX as an anti-diabetic agent in the future [34]. DM causes various complications in multiple organs [1,10,15,17]. Long-term hyperglycemia is associated with impaired BM retention and functional defects in BM-derived stem cells, eventually resulting in abnormal hematopoiesis and bone mass accrual [9,11,35]. Similar to a previous report [31], the current findings indicate that the function and BM retention of MSCs are predominantly impaired compared with those of HSCs. It is demonstrated that trabecular bone is more impaired than cortical bone by hyperglycemic conditions. This indicates that long-term hyperglycemia disrupts specialized BM niches and the hematopoietic system, thereby promoting diabetes-associated mortality. The present findings also indicate that diabetes-induced impairments in BM and hematopoietic and mesenchymal stem/stromal cells in the bone are recoverable via long-term supplementation with ASTX, although the recovery in function is not to baseline level.

ROS accumulation and subsequent oxidative damage are thought to be the main causes of diabetes-related complications; investigators have therefore focused their efforts on developing diabetes-specific antioxidant therapies. Ohshima et al. reported that antioxidants attenuate diabetes-induced disorders, such as cellular ROS accumulation and dysregulated BM retention of stem cells [22]. In addition to direct ROS scavenging capacity, antioxidants control cellular redox balance and stimulate the antioxidant defense system. Various biomolecules contribute to the maintenance of a balanced cellular redox state and modulate the antioxidant defense system in response to intra- and/or extra-cellular stimuli. In this regard, Nrf2 is considered a master transcriptional regulator of the antioxidant defense system and protects against oxidative stress-mediated impairments in the BM microenvironment and the functions of BM-derived stem cells [36,37]. There is also accumulating evidence that Nrf2 and Nrf2-dependent signaling exert important roles in protecting against diabetes-accompanied chronic complications [38,39,40,41]. Moreover, Nrf2 is thought to be the main molecular therapeutic target of ASTX [4,28,29,42]. Taken together, the current data, along with those of previous studies, indicate that long-term oral gavage of ASTX inhibits STZ-induced impairments in the functions of BM and BM-retained cells, as well as in hematopoietic development and bone homeostasis. Overall, the current study with the previous report [29] suggest that these effects of ASTX are orchestrated by its anti-oxidative activity along with the recovery of the Nrf2-related antioxidant defense system.

The maintenance, self-renewal, and peripheral migration of BM cells are tightly regulated by several signaling pathways. Wnt signaling plays crucial roles in embryogenesis, tissue development, homeostasis [43], and the maintenance and self-renewal of BM HSCs [44,45]. SDF-1 is an essential factor in CXCR4-linked maintenance and survival of BM HSCs and MSCs [46,47]. The Ang1/Tie2 signaling axis controls angiogenesis and hematopoiesis [31]. Therefore, dysregulation of these signaling pathways could impair BM retention and senescence of hematopoietic and mesenchymal stem/stromal cells in the bone, resulting in abnormal hematopoiesis and bone mass accrual. These results indicate that STZ-induced diabetes disrupts the BM microenvironment and hematopoietic development by decreasing the expression of several key ligands, such as SDF-1, Ang1, and β-catenin in the BM. Further experiments are needed to clarify whether the ASTX-mediated recovery in peripheral blood cell production and bone mass accrual in STZ-injected mice is directly associated with its ability to restore cellular levels of these ligands or is a concomitant event that results from its anti-oxidative and anti-inflammatory effects.

While long-term hyperglycemia causes chronic oxidative and inflammatory complications in various organs, diabetes-mediated death is correlated with the impaired retention, function, and peripheral circulation of BM HSCs [48]. The current findings indicate that supplemental ASTX increases the lifespan of STZ-injected mice, and this is closely associated with recovery from diabetes-induced BM complications, such as impaired retention and senescence of hematopoietic cells and abnormal production of circulating blood cells. IHC results in this study also indicate that STZ-induced destruction of pancreatic islets is in part recoverable via long-term supplementation with ASTX. However, based on the systemic complications observed in the STX+ASTX group, oral gavage of ASTX does not directly improve insulin secretion and sensitivity. In contrast, supplemental ASTX tended to ameliorate hyperglycemia-triggered chronic oxidative complications by recovering BM retention and the functions of HSCs and MSCs, as well as by restoring the Nrf2-related antioxidant defense system [23,24,25,26,27,28,49]. Considering previous findings showing that ASTX treatment alone does not alter enzyme activities of lymphocytes [22] and number of MitoSox-positive human-derived periodontal ligament fibroblasts [31], it is considered that supplementation with ASTX alone may not directly affect the functions of MSCs and hematopoietic development.

## 5. Conclusions

This study shows that long-term oral gavage of ASTX protects against STZ-induced damage to the BM microenvironment and the functions of BM HSCs and MSCs. This study also suggests that supplemental ASTX ameliorates STZ-induced hematopoietic development and bone mass accrual in mice, and that this is closely associated with its ability to inhibit oxidative damage and maintain the Nrf2-related antioxidant defense system. Overall, the current findings highlight the clinical potential of ASTX in ameliorating diabetes-induced oxidative complications in various organs and preventing excessive induction of stem cell senescence.

## Data Availability

The data presented in this study are available in the article and Supplementary Materials.

## Supplementary Materials

The following supporting information can be downloaded at: https://www.mdpi.com/article/10.3390/antiox11122321/s1, Figure S1: Supplemental ASTX does not ameliorate STZ-induced loss of body weight in STZ-injected mice, Figure S2: Supplemental ASTX suppresses hyperglycemia-induced oxidative stress in BM-derived LSK cells and HSCs of STZ-injected mice, Figure S3: STZ injection or in combination with ASTX does not increase levels of BM MSCs positive to MitoSox or DCF in mice, Table S1: Sequences of primers used for qRT-PCR.
