# Supplementary material for: Astaxanthin Protects against Hyperglycemia-Induced Oxidative and Inflammatory Damage to Bone Marrow and to Bone Marrow-Retained Stem Cells and Restores Normal Hematopoiesis in Streptozotocin-Induced Diabetic Mice
Source: Antioxidants (Basel). 2022 Nov 23;11(12):2321. doi: 10.3390/antiox11122321 (PMC9774236; doi:10.3390/antiox11122321)
Supplement: Supplementary file 1 [file antioxidants-11-02321-s001.zip › antioxidants-2004033-supplementary.pdf]

Supporting Information

Table S1. Sequences of primers used for qRT-PCR.

**Genes**	**Forward Primers**	**Reverse Primers**	**NCBI Reference Sequence Number**
Ang1	cagcacgaaggatgctgataac	ttgtcccgcagtgtagaacatt	NM_001286062.1
β-Catenin	ccgttcgccttcattatgga	ggcaaggtttcgaatcaatcc	NM_001165902.1
DKK1	caaaaatgtatcacaccaaaggacaa	tgcttggtacacacttgaccttctt	NM_010051.3
CXCR4	cctgcccggcatcgtcatcc	tgcgcttctggtggcccttg	NM_009911.3
SDF-1	tggggcctctgggcacagtt	aagccctgcaagcgttcctcg	NM_001012477
NFATc1	ggtgccttttgcgagcagtatc	cgtatggaccagaatgtgacgg	NM_001164109.1
TRAP	gatgactttgccagtcagca	acatagcccacaccgttctc	NM_007388.3
CTSK	ggccaactcaagaagaaaac	gtgcttgcttcccttctgg	NM_007802.4
GAPDH	gacggccgcatcttcttg	cacaccgaccttcaccat	XM_017321385.1

Supplemental figures and legends

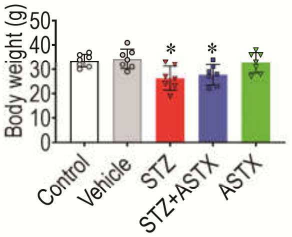

Figure S1. Supplemental ASTX does not ameliorate STZ-induced loss of body weight in STZ-injected mice. Body weight in mice groups were measured 60 days after hyperglycemia induction ($n = 7$). $^{*}p < 0.05$ by unpaired Student *t*-test.

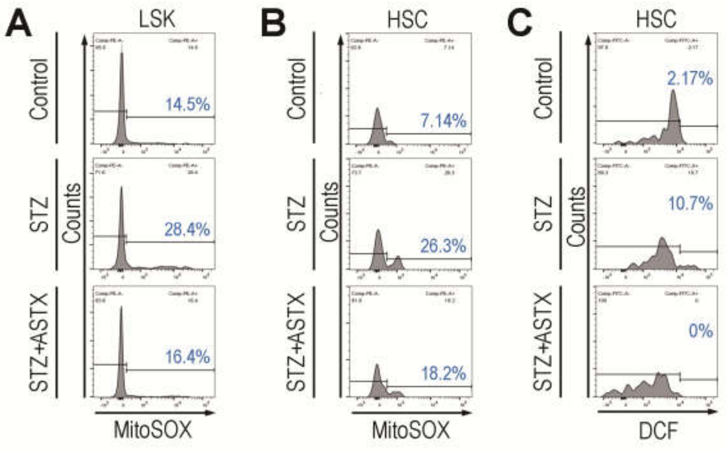

Figure S2. Supplemental ASTX suppresses hyperglycemia-induced oxidative stress in BM-derived LSK cells and HSCs of STZ-injected mice. (**A**,**B**) MitoSox- or (**C**) DCF-specific mean intensities in BM-derived LSK cells and HSCs of mice groups were determined by flow cytometry at 60 days post-hyperglycemia induction. A representative result from five different samples is shown.

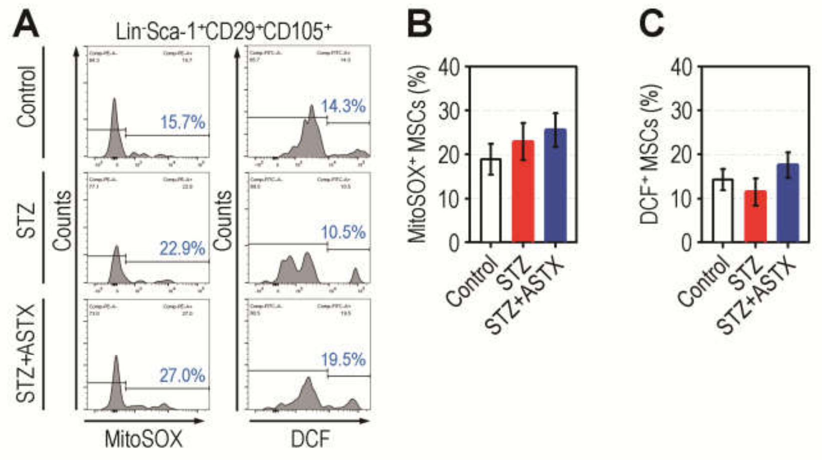

Figure S3. STZ injection or in combination with ASTX does not increase levels of BM MSCs positive to MitoSox or DCF in mice. (**A**) Flow cytometric analysis exhibiting the MitoSox- or DCF-specific mean intensities in BM-derived MSCs of mice groups at 60 days post-hyperglycemia induction. The percentages of (**B**) MitoSox^{+} and (**C**) DCF^{+} MSCs were calculated (n = 5).
